# Akt mediated phosphorylation of LARP6; critical step in biosynthesis of type I collagen

**DOI:** 10.1038/srep22597

**Published:** 2016-03-02

**Authors:** Yujie Zhang, Branko Stefanovic

**Affiliations:** 1 Department of Biomedical Sciences, College of Medicine, Florida State University, Tallahassee, Florida 32306, USA

## Abstract

La ribonucleoprotein domain family, member 6 (LARP6) is the RNA binding protein, which regulates translation of collagen mRNAs and synthesis of type I collagen. Posttranslational modifications of LARP6 and how they affect type I collagen synthesis have not been studied. We show that in lung fibroblasts LARP6 is phosphorylated at 8 serines, 6 of which are located within C-terminal domain. Phosphorylation of LARP6 follows a hierarchical order; S451 phosphorylation being a prerequisite for phosphorylations of other serines. Inhibition of PI3K/Akt pathway reduced the phosphorylation of LARP6, but had no effect on the S451A mutant, suggesting that PI3K/Akt pathway targets S451 and we have identified Akt as the responsible kinase. Overexpression of S451A mutant had dominant negative effect on collagen biosynthesis; drastically reduced secretion of collagen and induced hyper-modifications of collagen α2 (I) polypeptides. This indicates that LARP6 phosphorylation at S451 is critical for regulating translation and folding of collagen polypeptides. Akt inhibitor, GSK-2141795, which is in clinical trials for treatment of solid tumors, reduced collagen production by human lung fibroblasts with EC_50_ of 150 nM. This effect can be explained by inhibition of LARP6 phosphorylation and suggests that Akt inhibitors may be effective in treatment of various forms of fibrosis.

Type I collagen is triple helical protein composed of two α1 (I) polypeptides and one α2 (I) polypeptide. The constitutive rate of collagen synthesis is low, because the protein is very stable with half life of 60 days^1^. Physiologically, type I collagen expression can be rapidly upregulated during wound healing, but excessive collagen expression results in fibrosis leading to organ failure^2^. Therefore, elucidating the mechanism of type I collagen expression is important to our understanding of wound healing, pathogenesis of fibrosis and developing of anti-fibrotic drugs.

The biosynthesis of type I collagen is involving translation of the individual polypeptides, their posttranslational modifications, folding of three polypeptides into a triple helix, secretion of the triple helix into the extracellular space and processing of the secreted procollagen into mature collagen^3^,^4^. Expression of collagen polypeptides is regulated at transcriptional and post-transcriptional level^5^,^6^,^7^,^8^. Compelling evidence has been presented that posttranscriptional regulation plays important role in collagen expression^5^,^6^,^9^,^10^,^11^,^12^,^13^,^14^,^15^. Posttranscriptional regulation involves stabilization of collagen mRNAs and regulation of their translation and is mediated by two RNA binding proteins; αCP^16^ and La ribonucleoprotein domain family, member 6 (LARP6)^17^. αCP stabilizes collagen α1(I) mRNA by interacting with the cytosine-rich sequence in the 3′ untranslated region (3′UTR), and prolongs the half life of collagen mRNA^7^,^18^. LARP6 regulates stability, subcellular localization and translation of collagen mRNAs by binding a secondary structure found in the 5′ UTR of collagen α1(I) and α2(I) mRNAs^5^,^6^,^9^,^17^. In the 5′ UTR of these mRNAs, there is an evolutionary conserved 5′ stem-loop (5′SL) structure^19^. LARP6 binds the 5′SL with high affinity and specificity and functions as collagen mRNA specific adapter protein to tether the effectors of translation^20^. LARP6 interacts with nonmuscle myosin^21^, vimentin^6^, RNA helicase A (RHA)^11^, and serine-threonine kinase receptor-associated protein (STRAP)^10^. The interaction with myosin and STRAP coordinates translation of α1 (I) mRNA to that of α2 (I) mRNA, the interaction with RHA increases translational competitiveness of collagen mRNAs, while interaction with vimentin filaments prolongs their half life. In addition, subcellular partitioning of collagen mRNAs to the ER membrane depends on LARP6 and on integrity of nonmuscle myosin filaments^5^.

Collagen polypeptides are cotranslationally inserted into the ER lumen. During this process nascent polypeptides acquire several modifications, including hydroxylations of selected lysines and prolines and glycosylation of hydroxyl-lysine residues. Then, two pro α1(I) and one pro α2(I) chains associate at the carboxyl terminus and initiate folding into triple helix, which is propagated towards the amino terminus in a zipper-like fashion to form a procollagen molecule^4^,^22^,^23^. Modification and folding of collagen polypeptides are in dynamic equilibrium and slow folding results in hyper-modification of the polypeptides. Hyper-modified collagen polypeptides are unstable and phenotypically manifested as brittle bone disease, osteogenesis imperfecta^24^,^25^. The role of LARP6 in coupling translation of type I collagen polypeptides to their proper posttranslational modifications has been reported^5^,^21^.

There have been no reports on phosphorylation of LARP6 in the literature. The aim of this study was to determine if LARP6 is phosphorylated, to identify the phosphorylation sites, the kinase(s) involved and the role of LARP6 phosphorylation in type I collagen expression. We report that LARP6 is phosphorylated at multiple serines and that phosphorylation of S451 by Akt is critical for its regulation of type I collagen expression. We also propose a novel mechanism for the anti-fibrotic effect of Akt inhibition.

## Results

### Identification of LARP6 phosphorylation sites

**Figure 1:**
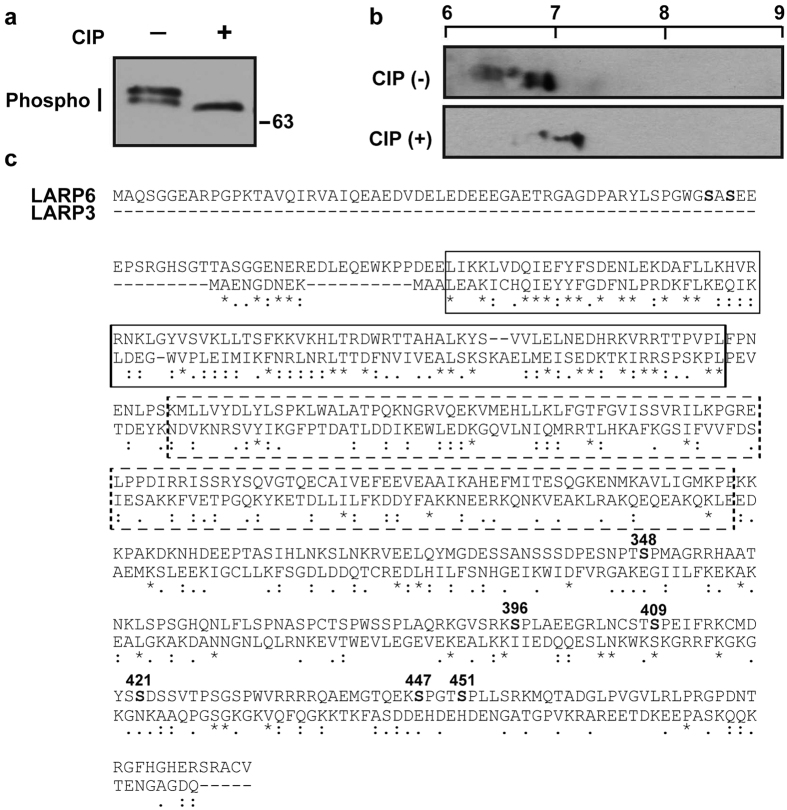
LARP6 as phosphoprotein. (**a**) Phosphorylated isoforms of LARP6 resolved by one dimension SDS PAGE (1DGE). Cell lysates of HLFs were incubated with or without calf intestinal alkaline phosphatase (CIP), resolved by 1DGE and endogenous LARP6 was visualized by Western blotting. (**b**) Phosphorylated isoforms of LARP6 resolved by 2DGE. Top panel: cell lysates were resolved by isoelectric focusing in the first dimension and by SDS PAGE in the second dimension and endogenous LARP6 was visualized by Western blotting. Bottom panel: same extract after treatment with CIP. pH scale is shown on the top. (**c**) Alignment of amino acid sequence of human LARP6 and LARP3. La homology domain is marked by full box and RNA recognition motif by dashed line box. The phosphorylated amino acids identified at the carboxyl terminus are indicated in bold. *indicate identities and dots indicate similarities between LARP6 and LARP3.

To assess if LARP6 is phosphorylated, we analyzed LARP6 migration in SDS-PAGE gels. In one dimensional SDS-PAGE (1DGE) LARP6 from human lung fibroblasts (HLFs) was resolved as two bands, which were reduced to a single, faster migrating, band after calf intestinal alkaline phosphatase (CIP) treatment (Fig. 1a), suggesting that the two bands represent phosphorylated forms of LARP6. To assess the extent of phosphorylations the isoelectric point of LARP6 was analyzed by two dimensional electrophoresis (2DGE; isoelectric focusing (IEF) followed by SDS-PAGE and Western blotting) (Fig. 1b). LARP6 was resolved as series of molecules having the pI between 6.2–7.0, suggesting that LARP6 exists as a series of isoforms carrying different charges. After CIP treatment a reduction in the abundance of the acidic isoforms and accumulation of a major isoform with pI of 7.2 was observed, indicating dephosphorylation of LARP6 (Fig. 1b). The theoretical pI of non-phosphorylated LARP6 is 8.4, however, after treatment with CIP LARP6 had pI of about 7.2. The discrepancy between the predicted and observed pI may be due to incomplete digestion of LARP6 by CIP or inaccurate pH calibration of the isoelectric focusing strips. We have observed that different batches of strips produce results that can differ by as much as 1 pH unit. Therefore, throughout this manuscript we only compared the pI of proteins when they had been resolved on the same batch of strips. Taken together, we concluded that LARP6 is phosphorylated at multiple sites.

To determine which amino acids are modified by phosphorylation, we analyzed the phospho-peptides by Mass Spectrometry. In three independent experiments 8 phosphorylation sites on LARP6 were identified; they are Ser56, Ser58, Ser348, Ser396, Ser409, Ser421, Ser447, and Ser451 (Table 1). Identification of 8 phosphorylation sites is consistent with the range of pI values of endogenous LARP6 revealed by 2DGE (Fig. 1b).

The sequence comparison between LARP3 and LARP6 is shown in Fig. 1c. LARP6 contains four domains: the amino terminal domain, the La homology domain (LA domain) (full box), an RNA recognition motif (RRM) (dashed line box), and the carboxyl terminal domain^26^. The LA domain and RRM together are needed for binding 5′SL of collagen mRNAs^9^, while the carboxyl terminal domain is involved in interactions with other proteins, such as STRAP, nonmuscle myosin, and FKBP3^10^,^21^,^27^. This domain is also highly divergent from LARP3 and other LARP superfamily members^28^ and all of the phospho-serine residues in this domain, except S409, do not have a counterpart in LARP3. We hypothesized that phosphorylations of LARP6 within the carboxyl terminal domain are important for its specific role in regulating translation of type I collagen mRNAs.

### Phosphorylation of Ser451 by PI3K/Akt signaling pathway as primary event in posttranslational modifications of LARP6

**Figure 2:**
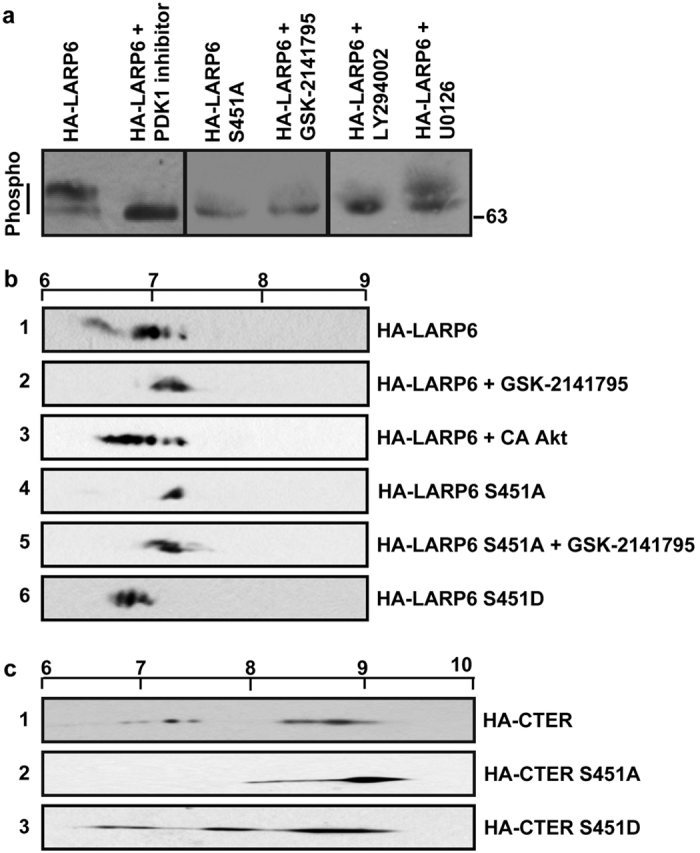
Identification of kinase involved in phosphorylation of S451 of LARP6. (**a**) Inhibition of LARP6 phosphorylation by kinase inhibitors. HLFs were pretreated with the indicated kinase inhibitors, wt HA-LARP6 or S451A mutant were expressed in the treated cells and proteins were analyzed by 1DGE and Western blotting. Cropped gels were run under the same experimental conditions, and indicated by vertical lines. (**b**) Isoelectric focusing of HA-LARP6 analyzed by 2DGE and Western blotting. Panel 1: wt HA-LARP6 expressed in HEK293 cells. Panel 2: wt HA-LARP6 in HEK293 cells treated with Akt inhibitor, GSK-2141795. Panel 3: wt HA-LARP6 in HEK293 cells co-expressing CA Akt. Panel 4: S451A mutant of HA-LARP6 expressed in HEK293 cells. Panel 5: S451A mutant in cells treated with Akt inhibitor, GSK-2141795. Panel 6: S451D mutant expressed in HEK293 cells. The scale at the top indicates pH. (**c**) Phosphorylation of the CTER of LARP6. HA-CTER of LARP6 (panel 1), HA-CTER having S451A mutation (panel 2) or CTER with S451D mutation (panel 3) were expressed in HEK293 cells and analyzed by 2DGE. The scale on the top indicates pH.

To confirm that the serines identified by Mass Spectrometry are phosphorylated we individually mutated the 6 serines within the C-terminal domain into alanines and subjected the mutants to 2DGE analysis (isoelectric focusing (IEF) followed by SDS-PAGE and Western blotting). The rationale was that if a serine is phosphorylated, its mutation into alanine will result in a loss of a negative charge, which can be scored as a shift of the pI spectrum into the more basic region of the isoelectric focusing strip. Consequently, if there is a gain of negative charge by phosphorylation the pI values will be shifted to the more acidic region. Figure 2b shows the 2DGE analysis of HA-tagged full size LARP6 expressed in HLFs. The phosphorylation analysis of wt HA-LARP6 in nontreated cells showed molecular species (dots on 2DGE) with the pI between 6.8 and 7.4, revealing the spectrum of LARP6 phosphorylation states (Fig. 2b, panel 1). Mutations of the individual S348, S396, S409, S421 and S447 caused small alteration of this spectrum, consistent with loss of a single negative charge (not shown). However, when S451 was changed into alanine, a dramatic change in the pI was observed, with loss of at least 3 phospho signals (dots on 2DGE) (Fig. 2b, compare panels 1 and 4). The S451A mutant was isoelectrically focused as homogeneous molecular species with pI of 7.3 (Fig. 2b, panel 4); this is in sharp contrast to the pI of wt HA-LARP6 which showed 3-4 phospho signals over pH range of 6.8–7.4 (Fig. 2b, panel 1). This result suggests that several phosphorylation events had been abolished and not just one and we concluded that the S451A mutation prevented, not only the phosphorylation at this serine, but also additional phosphorylation events. This is further verified when phosphorylations of the isolated C-terminal domain of LARP6 (CTER) were analyzed (see later, Fig. 2c). The phospho-mimetic mutation of S451 (S451D) restored the normal pattern of LARP6 phosphorylation, including appearance LARP6 molecules with full spectrum of the pI values (Fig. 2b, compare panels 6 and 1), consistent with the notion that Ser451 must be phosphorylated for other modifications to take place.

Based on the importance of S451 phosphorylation, the next step was to identify the kinase involved. We surmised that, if S451 phosphorylation is required for acquisition of other phosphorylations, inhibition of the kinase involved in phosphorylation of S451 would result in drastic hypophosphorylation of LARP6, which could be detected in 1DGE (see Fig. 1a). Therefore, we pre-treated HLFs with various kinase inhibitors, transfected HA-LARP6 and used 1DGE to assess the change in electrophoretic mobility of the protein. In control cells HA-LARP6 was resolved as two bands, as before, representing phosphorylated LARP6 (Fig. 2a, lane 1). The transfected S451A mutant migrated as a single band, consistent with the notion that this mutant has lost most of its phosphorylations (Fig. 2a, lane 3). Then, we assessed which kinase inhibitor will render the electrophoretic mobility of wt HA-LARP6 similar to that of the S451A mutant. We screened 19 kinase inhibitors and only PI3K inhibitor (LY294002)^29^, PDK1 inhibitor^30^ and Akt inhibitor (GSK-2141795)^31^, reduced the migration of wt HA-LARP6 to a single band (Fig. 2a, lanes 2, 4 and 5). As control, we show that MEK1/2 inhibitor (U0126)^32^,^33^,^34^ had no effect on electrophoretic mobility of wt HA-LARP6 (Fig. 2a, lane 6). All three kinases identified participate in the PI3K/Akt signaling pathway, with PI3K and PDK1 acting upstream of Akt, suggesting that PI3K/Akt pathway is involved in LARP6 phosphorylation at S451.

To provide additional evidence that PI3K/Akt signaling pathway phosphorylates LARP6 we analyzed the pI of HA-LARP6 after treatment of Akt inhibitor, GSK-2141795. The inhibitor shifted the pI of HA-LARP6 molecules from 6.8–7.4 (Fig. 2b, panel 1) to 7.3–7.4 (Fig. 2b, panel 2). However, it failed to change the pI of the S451A mutant (Fig. 2b, compare panels 4 and 5), suggesting that this mutant has lost its responsiveness to the Akt dependent phosphorylation.

When constitutive active Akt (CA Akt)^35^ was co-expressed with wt HA-LARP6, we observed a shift of the pI into a more acidic region (Fig. 2b, panel 3), suggesting that LARP6 becomes hyperphosphorylated when CA Akt is present.

### C-terminal domain of LARP6 undergoes phosphorylations independently of the other parts of the protein

Because most of the phosphorylation sites were identified in the carboxyl terminal domain of LARP6, predicted to be unstructured, we assessed if this domain can undergo phosphorylations independently of the other domains of LARP6. 2DGE showed that HA-CTER was resolved with pI spectrum of 6.6–9.0 with multiple phospho signals, consistent with multiple phosphorylations (Fig. 2c, panel 1). The predicted pI of unphosphorylated HA-CTER is 9.21. The S451A mutant of HA-CTER was resolved as single predominant molecular species with pI around 9, with the absence of 3-4 phospho signals seen in wt HA-CTER (Fig. 2c, compare panels 1 and 2). This suggested that this mutant failed to acquire multiple phosphorylations and not only one. The pI spectrum of the HA-CTER S451D mutant was similar to that of wt HA-CTER (Fig. 2c, compare panels 1 and 3), demonstrating that this phospho-mimetic mutation rescued normal pI spectrum of LARP6 molecules. These results with C-terminal domain of LARP6 further corroborate the hypothesis that phosphorylation of S451 is the prerequisite for other phosphorylations to take place. Thus, we concluded that: 1. CTER can be phosphorylated independently of other parts of the protein; this means also independently of binding collagen mRNAs, 2. a hierarchical phosphorylation of the C-terminal domain of LARP6 is evident in which Ser451 phosphorylation is the priming event.

### Akt dependent LARP6 S451 phosphorylation *in vitro*

**Figure 3:**
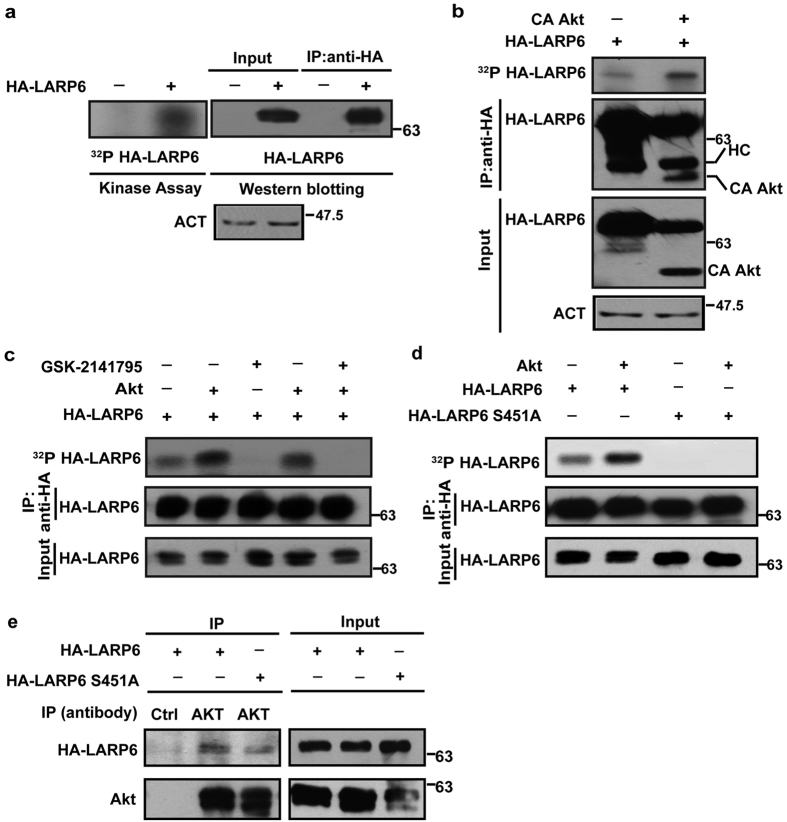
Akt dependent phosphorylation of LARP6. (**a**) Phosphorylation of HA-LARP6 after immunoprecipitation. Left panel: Immunoprecipitate of control HLFs (lane 1) and HLFs overexpressing HA-LARP6 (lane 2) was incubated with [γ-^32^P]ATP, followed by SDS PAGE and radioautography. Right panel: HA-LARP6 in the immunoprecipitate (lanes 3 and 4) or 10% of input (lanes 1 and 2) analyzed by Western blotting on the same gel as in the left panel. ACT: β-actin loading control for the input samples. (**b**) Increased phosphorylation of LARP6 immunoprecipitated from HEK293 cells co-expressing CA Akt. Top panel: HA-LARP6 immunoprecipitated from control HEK293 cells (lane 1) or from HEK293 cells co-expressing HA-tagged CA Akt (lane 2) and immunoprecipitate was incubated with [γ-^32^P]ATP, followed by SDS PAGE and radioautography. Middle panel: HA-LARP6 and CA Akt in the immunoprecipitate analyzed by Western blotting. HC, antibody heavy chain. Bottom panel: HA-LARP6 and CA Akt in 10% of input material. ACT: β-actin loading control for the input samples. (**c**) Akt inhibitor, GSK-2141795, abolishes *in vitro* phosphorylation of HA-LARP6. Top panel: HA-LARP6 was immunoprecipitated from HLFs and immunoprecipitate supplemented with [γ-^32^P]ATP and buffer (lane 1), purified active Akt enzyme (lanes 2, 4 and 5), GSK-2141795 (0.1 μM, lanes 3 and 5) and radio-labeling of LARP6 was analyzed by SDS PAGE and autoradiography. Middle and bottom panels: HA-LARP6 in the immunoprecipitate and in 10% of input analyzed by Western blotting. (**d**) Lack of phosphorylation of S451A mutant. Top panel: wt HA-LARP6 (lanes 1 and 2) or HA-tagged S451A mutant of LARP6 (lanes 3 and 4) were immunoprecipitated from HLFs. Purified active Akt was added in lanes 2 and 4 and *in vitro* kinase assay was done as in (**c**). Middle and bottom panels: HA-LARP6 in the immunoprecipitate and in 10% of input analyzed by Western blotting. (**e**) Interaction of LARP6 and Akt. Left panel: wt HA-LARP6 (lanes 1 and 2) and S451A mutant (lane 3) were expressed in HLFs. Immunoprecipitation was done with anti-Akt antibody (lanes 2 and 3) or control antibody (lane 1) and the pulled down material analyzed by Western blotting using anti-HA antibody. Expression of proteins in 10% of input material is shown in the right panel.

*In vitro* kinase assay was used to corroborate that Akt is involved in phosphorylation of LARP6 at serine 451. As substrate, LARP6 had to be immunoprecipitated after expression in mammalian cells. In the immunoprecipitate containing HA-LARP6 radiolabeling of the protein was observed (Fig. 3a, left panel, lane 2), which was absent from control immunoprecipitate lacking HA-LARP6 (Fig. 3a, left panel, lane 1). When the same samples were analyzed by Western blotting, HA-LARP6 was visualized as the protein with identical electrophoretic mobility as the radiolabeled band in the kinase assay (Fig. 3a, right panel, lanes 2 and 4). This verified that the protein radiolabeled in the immunoprecipitate was LARP6 and that the immunoprecipitate contained a kinase responsible for its phosphorylation.

To investigate if overexpression of Akt will increase LARP6 phosphorylation in the immunoprecipitate, we overexpressed HA-LARP6 with and without CA Akt. In these experiments CA Akt was also tagged with HA tag, so immunoprecipitation with anti-HA antibody pulled down both, LARP6 and CA Akt. When the immunoprecipitate without co-expression of CA Akt was incubated with [γ-^32^P]ATP a weak phosphorylation of HA-LARP6 was seen (Fig. 3b, top panel, lane 1). However, co-expression of CA Akt increased the phosphorylation of HA-LARP6 (Fig. 3b, top panel, lane 2). These results suggested that either the endogenous kinase present in the precipitate might have been Akt (the evidence for this is presented in Fig. 3e) and that expression of CA Akt increased the total Akt activity in the immunoprecipitate or that other kinase was pulled down with HA-LARP6 and that CA Akt might have stimulated its activity.

To provide additional evidence that Akt is involved in phosphorylation of S451, two types of experiments were performed; addition of Akt inhibitor GSK-2141795 to the immunoprecipitate and addition of pure active Akt protein to the immunoprecipitate. Again, weak HA-LARP6 phosphorylation was observed in the absence of adding Akt protein to the precipitate (Fig. 3c, lane 1). Addition of the purified Akt kinase to the precipitate increased the HA-LARP6 phosphorylation (Fig. 3c, lanes 2 and 4). The phosphorylation in the absence or presence of exogenous Akt protein was abolished by preincubation of the immunoprecipitate with Akt inhibitor, GSK-2141795 (Fig. 3c, lanes 3 and 5). Thus, the results corroborated that Akt participates in phosphorylation LARP6.

To verify that Akt dependent phosphorylation targets S451, we repeated the experiments using S451A mutant. In contrast to wt HA-LARP6 (Fig. 3d, lanes 1 and 2), phosphorylation of the S451A mutant was undetectable either with or without addition of purified Akt kinase to the precipitate (Fig. 3d, lanes 3 and 4). This strongly suggested that Akt dependent phosphorylation of LARP6 targets S451.

### Interaction of LARP6 and Akt

The presence of Akt activity in the immunoprecipitate could be explained if LARP6 and Akt form a complex. As shown in Fig. 3e, left panel, lane 2, endogenous Akt pulled down HA-LARP6, indicating that the two proteins co-immunoprecipitate. The S451A mutant was also immunoprecipitated with Akt, albeit with lower efficiency (Fig. 3e, left panel, compare lanes 2 and 3). This mutant showed undetectable phosphorylation in the immunoprecipitate (Fig. 3d), suggesting that the negative reaction was due to absence of the phosphorylation site rather than to its inability to interact with Akt. This also suggests that interaction of Akt and LARP6 is phosphorylation independent and that unphosphorylated LARP6 can be the Akt substrate. These results are consistent with a notion that LARP6 and Akt can interact and that pull down of HA-LARP6 can coprecipitate Akt.

### Inhibition of Akt reduces secretion of type I collagen

The hallmark of deregulated translation of collagen mRNAs is reduced secretion of type I collagen and appearance of hyper-modified collagen polypeptides^5^,^10^. Collagen polypeptides are modified by hydroxylations of prolines and lysines, which are the major modifications providing H-bonding between the polypeptides when they are folded into the triple helix. Lysine hydroxylations are associated with masking of the positively charged lysine NH_2_ groups^36^, causing a shift of the pI into the more acidic region. Excessive posttranslational modifications of individual polypeptides take place if the strict coordination of translation and folding of α1(I) and α2(I) polypeptides is not maintained. This coordination is regulated by LARP6^5^,^10^,^11^,^21^ and appearance of excessively modified collagen polypeptides can serve as a readout of perturbed LARP6 function. Therefore, if Akt dependent phosphorylation of LARP6 on S451 is functionally important, then the inhibition of Akt must affect secretion and modifications of type I collagen. Therefore, we analyzed both of these readouts.

**Figure 4:**
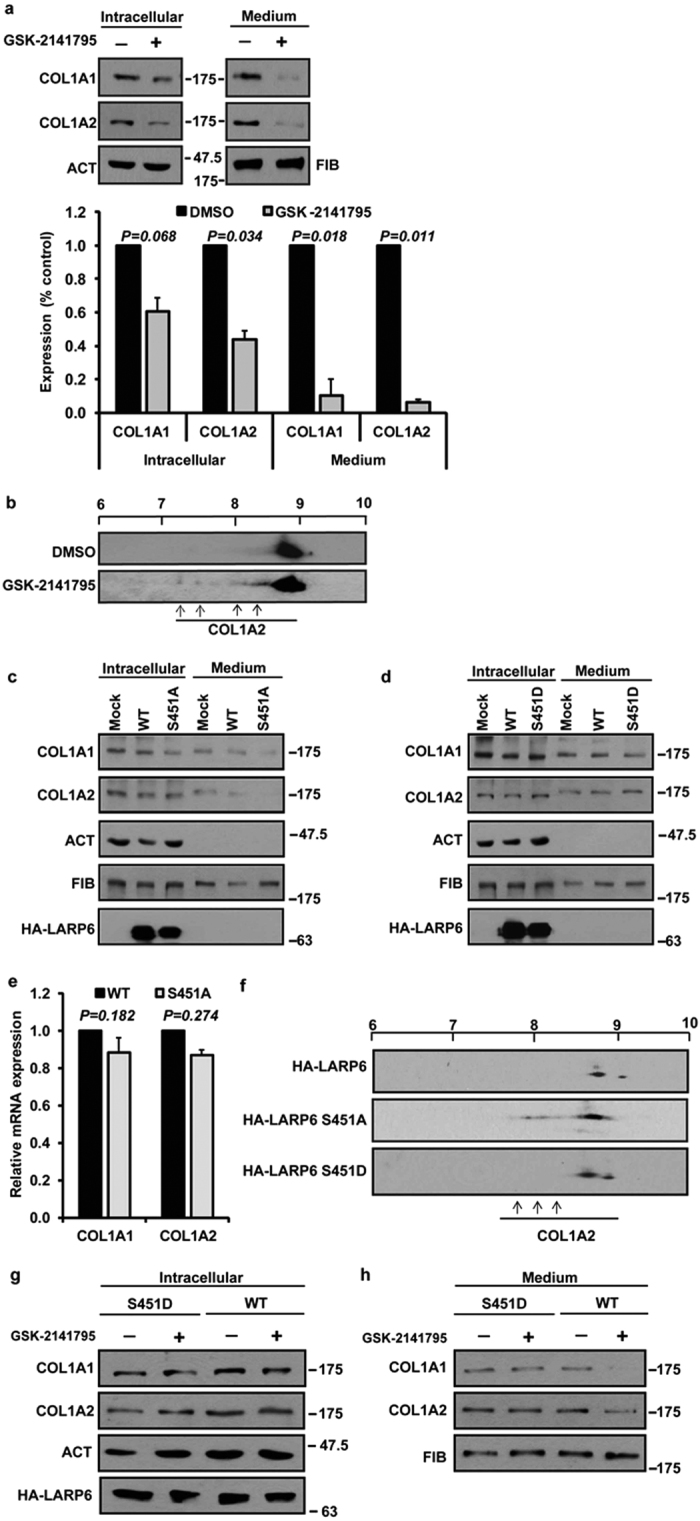
Reduced secretion of type I collagen by Akt inhibition and S451A overexpression. (**a**) Top panels: the level of collagen α1(I) (COL1A1) and collagen α2(I) (COL1A2) polypeptides in HLFs was analyzed intracellularly and in the cellular medium by Western blotting after DMSO (−) or Akt inhibiton by GSK-2141795 (+). Loading controls: β-actin (ACT) and fibronectin (FIB). Bottom panel: Western blots from 3 independent experiments as shown in top panels were quantified, normalized to β-actin (for intracellular collagen) and fibronectin (for medium collagen) and expressed as percentage of control cells. Error bars: standard deviation (SD) (n = 3). (**b**) Hyper-modifications of collagen α2(I) polypeptide after Akt inhibition analyzed by 2DGE and Western blotting. Hyper-modifications are indicated by arrows. The scale on the top indicates pH. (**c**) Dominant negative effect of S451A mutant on secretion of type I collagen. COL1A1 and COL1A2 polypeptides were measured in cellular extracts (lanes 1–3) and medium (lanes 4–6) of HLFs overexpressing wt HA-LARP6, S451A mutant or in mock transfected cells. Loading controls: β-actin (ACT) and fibronectin (FIB). HA-LARP6: expression of transfected proteins. (**d**) Same experiment as in (**c**), except S451D mutant was analyzed. (**e**) Expression of collagen mRNAs. Total RNA extracted from cells overexpressing wt and S451A LARP6 was analyzed for expression of COL1A1 and COL1A2 mRNAs by real-time PCR and normalized to β-actin mRNA. Error bars: SD (n = 3). (**f**) Modifications of collagen α2(I) polypeptide in cells overexpressing wt HA-LARP6, S451A or S451D mutant analyzed by 2DGE and Western blotting. Hyper-modifications are indicated by arrows and pH scale is on the top. (**g**) GSK-2141795 has no effect on cellular level of collagen polypeptides. HLFs were transfected with wt HA-LARP6 or S451D mutant and treated with DMSO (-) or GSK-2141795 (+) and collagen polypeptides (COL1A1 and COL1A2) were analyzed in cellular extracts by Western blotting. ACT: β-actin loading control. HA-LARP6: expression of transfected proteins. (**h**) Rescue of collagen secretion by S451D mutant. The medium from cells in (**g**) was analyzed for collagen polypeptides (COL1A1 and COL1A2) by Western blotting. FIB: fibronectin loading control.

When HLFs were treated with GSK-2141795, the intracellular level of collagen α1 (I) polypeptide was not significantly changed, while the level of α2 (I) polypeptide was slightly reduced. The densitometric scans of Western blots are shown in the bottom panel of Fig. 4a. However, the secretion of the both polypeptides into the cellular medium was drastically reduced (Fig. 4a); quantification of the Western blots showed ~10-fold reduction of both collagen polypeptides in the cellular medium (Fig. 4a, bottom panel).

We also analyzed modifications of collagen α2(I) polypeptide by 2DGE in cells treated with GSK-2141795. Failure of the available antibody to recognize α1(I) polypeptide after isoelectric focusing prevented the analysis of this polypeptide. In control cells the pI of the collagen α2(I) polypeptide was distributed over a narrow pI range, centered around 8.8, indicating presence of uniformly modified molecules (Fig. 4b, top panel). The predicted pI value of unmodified collagen α2 (I) polypeptide with cleaved off signal peptide is 9.2. When the cells were treated with GSK-2141795, a fraction of collagen α2(I) polypeptides with more acidic pI appeared, with pI extending from 7.2–8.0 (Fig. 4b, bottom panel, arrows). Based on this acidic shift in the pI, we concluded that α2(I) polypeptide underwent hyper-hydroxylations after inhibition of Akt. Overall, these results suggest that Akt inhibition results in poor secretion of type I collagen and uncoupling of translation and posttranslational modifications, resulting in over-modifications of the individual polypeptides. As these processes depend on LARP6, phosphorylation of LARP6 on S451 may serve to activate the protein in biosynthesis of type I collagen.

### S451A mutant of LARP6 acts as dominant negative protein in collagen synthesis

If S451 phosphorylation activates LARP6 to regulate type I collagen synthesis, then overexpression of S451A mutant should have similar effects on type I collagen as Akt inhibition. The intracellular level of collagen α1(I) and α2(I) polypeptide was similar in mock transfected cells and cells overexpressing wt or S451A HA-LARP6 (Fig. 4c, compare lanes 1, 2 and 3). Expression of collagen α1(I) and α2(I) mRNAs was also unaffected by overexpression of S451A mutant (Fig. 4e). However, the cells overexpressing the S451A mutant secreted type I collagen at reduced rate compare to mock transfected cells or cells overexpressing wt LARP6 (Fig. 4c, compare lanes 4, 5 and 6). In contrast to this dominant negative effect of S451A, when S451D mutant was overexpressed type I collage secretion was similar to that of mock transfected cells or cells overexpressing wt HA-LARP6 (Fig. 4d, compare lanes 4, 5 and 6). We concluded from these experiments that secretion of type I collagen can be reduced in two ways; by inhibiting Akt and by overexpressing S451A mutant of LARP6. This strongly argues that phosphorylation of S451 by Akt is a critical step in this process.

We also analyzed modifications of collagen α2 (I) polypeptide in cells overexpressing S451A by 2DGE. Overexpression of S451A mutant induced appearance of α2(I) molecules with more acidic pI, between 7.9 and 8.1 (Fig. 4f, middle panel, arrows). The accumulation of more acidic α2(I) polypeptides was also seen after inhibition of Akt (Fig. 4b), suggesting that both ways result in similar alteration of collagen biosynthesis. When S451D mutant was overexpressed, the pI of α2(I) polypeptide was identical to that in wt HA-LARP6 expressing cells (Fig. 4f, lower panel). Overall, these results support the hypothesis that phosphorylation of S451 by Akt is a necessary step to activate productive synthesis type I collagen and suggests that secretion and modifications of type I collagen are impaired if LARP6 is not phosphorylated on S451.

### Phospho-mimetic LARP6 mutant, S451D, rescues collagen expression after Akt inhibition

If the inhibition of Akt reduces secretion of type I collagen due to absence of S451 phosphorylation, then expression of the LARP6 S451D mutant should overcome this inhibitory effect. Therefore, we analyzed if the phospho-mimetic mutation, S451D, can rescue type I collagen secretion when Akt is inhibited. Inhibition of Akt by GSK-2141795 did not change the intracellular level of collagen α1(I) and α2(I) polypeptides in cells overexpressing either the S451D mutant or the wt HA-LARP6 (Fig. 4g). However, the inhibitor reduced secretion of type I collagen polypeptides from cells overexpressing wt HA-LARP6 (Fig. 4h, compare lanes 3 and 4), but not from the cells overexpressing S451D mutant (Fig. 4h, compare lanes 1 and 2). This result suggests that phospho-mimetic mutation of S451 can overcome the effect of Akt inhibition and corroborate the hypothesis that phosphorylation of S451 by Akt is a critical step to activate productive type I collagen synthesis.

### Effect of S451 phosphorylation on stability of LARP6 protein

**Figure 5:**
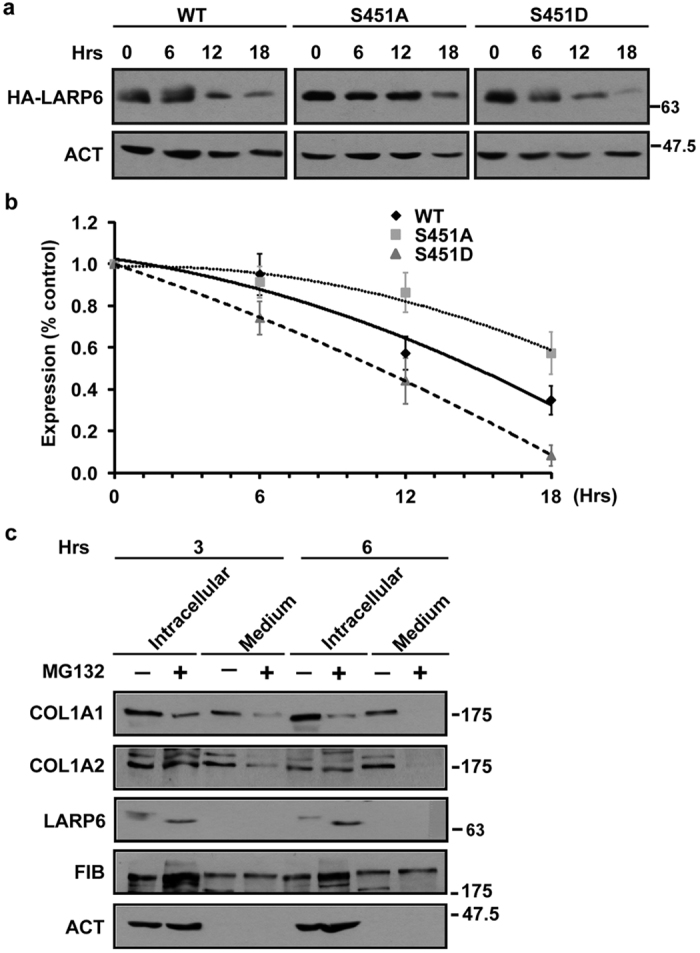
Regulation of LARP6 stability by phosphorylation. (**a**) Decay of wt HA-LARP6 and S451A and S451D mutants. HLFs expressing wt HA-LARP6 and S451A and S451D mutants were treated with cycloheximide for the indicated time points and protein level analyzed by Western blotting. ACT: β-actin loading control. (**b**) Decay rate of wt HA-LAPR6 and mutants. Western blots from 3 independent experiments as shown in (**a**) were quantified and normalized to β-actin level. Each data point is presented as the mean ± SD (n = 3). (**c**) Increased accumulation of LARP6 after proteasome inhibition is associated with reduced secretion of collagen polypeptides. HLFs were treated with DMSO (−) or MG132 (+) for 3 hours (lanes 1–4) and for 6 hours (lanes 5–8) and cellular level and secretion into the medium of collagen polypeptides (COL1A1 and COL1A2) was measured by Western blotting. LARP6: expression of endogenous LARP6. Loading controls: β-actin (ACT) and fibronectin (FIB).

While we have demonstrated that phosphorylation of S451 is a prerequisite for additional phosphorylations and for participation of LARP6 in collagen biosynthesis, we have also observed that the S451 phosphorylation regulates half life of the protein. wt HA-LARP6 decayed with a half life of 15.5 hours, S451D mutant had a half life of 11.5 hours and S451A mutant had a half life of >18 hours (Fig. 5a,b). This indicated that S451 phosphorylation increases the turnover of the protein and suggests that activation of LARP6 by S451 phosphorylation is coupled to its more rapid degradation.

To investigate if there is an association between the half life of LARP6 and collagen expression, we artificially prolonged the half life of endogenous LARP6 by treating HLFs with proteasome inhibitor, MG132. Between 3 hours and 6 hours of MG132 treatment we observed that LARP6 accumulated at the higher level in the cells, suggesting that its degradation has been retarded (Fig. 5c). When we compared collagen expression at 3 hours and 6 hours after the MG132 treatment, the intracellular level of collagen polypeptides was unaffected (Fig. 5c, compare lanes 2 and 6). However, at 6 hours secretion of both collagen polypeptides into the cellular medium was reduced when compared to the level seen at 3 hours (Fig. 5c, compare lanes 4 and 8). Although interpretation of these results may not be straightforward, because proteasome inhibition can have multitude of effects, the increased half life of LARP6 was associated with decreased collagen excretion. This points to the notion that proper turnover of LARP6 may be important for its function.

### Dose dependent reduction in type I collagen by Akt inhibitor, GSK-2141795

**Figure 6:**
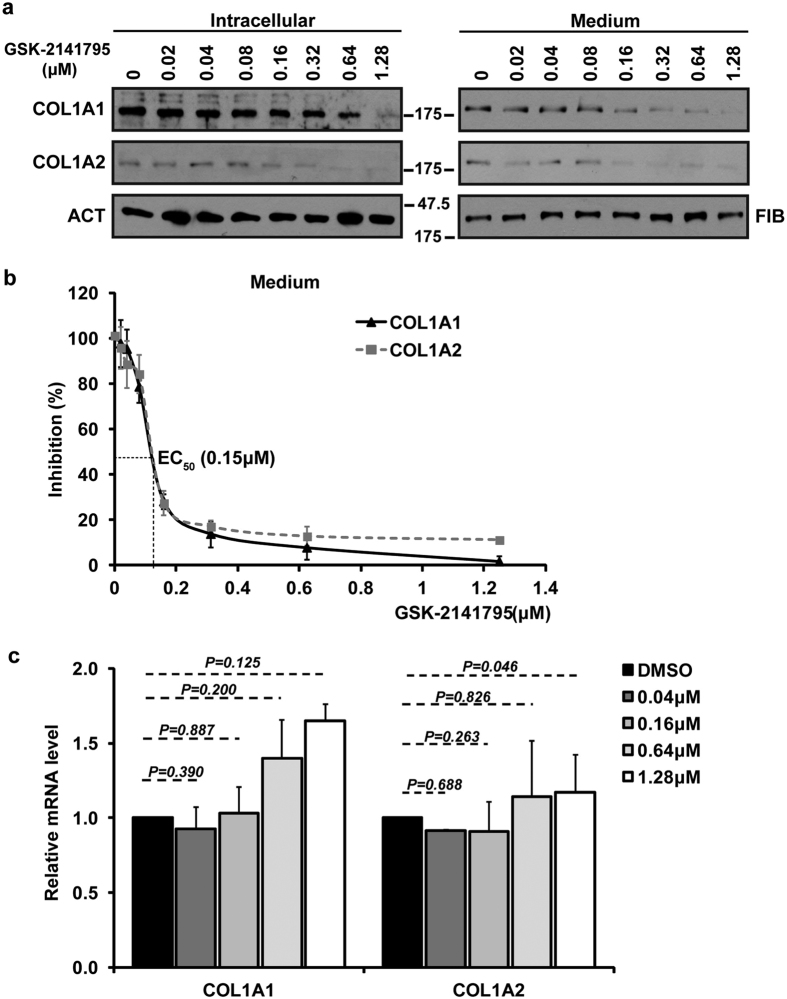
GSK-2141795 reduces secretion of type I collagen at concentrations that are inhibitory to Akt. (**a**) Intracellular levels (left panels) and secretion into the cellular medium (right panels) of COL1A1 and COL1A2 polypeptides was measured by Western blotting after treating HLFs with the indicated doses of GSK-2141795. ACT and FIB: β-actin and fibronection, as loading controls. (**b**) EC_50_ of GSK-2141795 induced inhibition of type I collagen secretion. Western blots from 3 independent experiments as shown in (a, right panels) were quantified, normalized to fibronectin and presented as percent of control cells. Error bars: SD (n = 3). (**c**) Expression of collagen mRNAs in GSK-2141795 treated cells. Total RNA from cells in (**a**) was analyzed for expression of COL1A1 and COL1A2 mRNAs by real-time PCR and normalized to β-actin mRNA. Error bars: SD (n = 3).

Akt inhibitor, GSK-2141795, has the IC_50_ for Akt1 of 180 nM, for Akt2 of 328 nM and for Akt3 of 38 nM^31^,^37^. This inhibitor is in clinical trials as an anticancer drug, but our results (Fig. 4) suggest that it may also have anti-fibrotic activity. To test if GSK-2141795 inhibits type I collagen production at the concentrations at which it inhibits various isoforms of Akt, we treated HLFs with GSK-2141795 in concentration range from 20 nM to 1.28 μM and analyzed type I collagen expression. At concentration of 320 nM or higher GSK-2141795 reduced intracellular level of collagen α2(I) polypeptide, while it reduced intracellular α1(I) polypeptide at concentrations >640 nM (Fig. 6a, left panels). These concentrations are above the IC_50_ for Akt isoforms and may reflect nonspecific effects seen at high concentrations of most inhibitors. However, GSK-2141795 reduced secretion of type I collagen at concentrations of 160 nM which is lower than IC_50_ for Akt1 and Akt2 (Fig. 6a, right panels). Quantitative analysis of type I collagen in the cellular medium revealed that the effective concentration of GSK-2141795 for 50% reduction (EC_50_) was about 150 nM (Fig. 6b). This indicates that GSK-2141795 affects secretion of collagen peptides at concentrations that inhibit Akt. We also measured the steady state levels of collagen mRNA and found that GSK-2141795 did not change their level at any concentration tested (Fig. 6c). Overall, these results provided the first evidence that this orally bioavailable Akt inhibitor, currently in phase II clinical trials for cancers, inhibits type I collagen production by inhibiting LARP6 phosphorylation.

## Discussion

We provide the first description that LARP6 is phosphorylated at multiple sites and that phosphorylation of S451 is critical to activate the protein in type I collagen biosynthesis. We find that: (i) LARP6 is phosphorylated at 8 serines, (ii) phosphorylation of LARP6 at S451 by Akt is required for phosphorylation of several other sites, (iii) the S451A mutant of LARP6 has dominant negative effect on type I collagen production by reducing secretion and causing hyper-modifications of α2 (I) polypeptide, (iv) similar effect can be induced by Akt inhibition, (v) Akt inhibitor GSK-2141795 reduces secretion of type I collagen at therapeutic concentrations. These results demonstrate that phosphorylation of LARP6 at S451 is an important step to activate LARP6 in collagen biosynthesis and to regulate translation and folding of type I collagen polypeptides.

This is also the first report on functional significance of phosphorylation of LARP6. Large scale mapping of phosphoproteins in A498 and Hela cells identified phosphorylation of LARP6 at S56 and S58^38^,^39^, but not the other phosphorylation sites identified here. The reason for this may be that collagen producing cells have not been included in these phosphoproteome studies. In HLFs we have identified 6 additional phosphorylation events at the C-terminal domain of LARP6. The C-terminal domain does not bind 5′SL of collagen mRNAs, so phosphorylations in this domain are not dependent on binding collagen mRNAs. However, the C-terminal domain is involved in interactions of LARP6 and other proteins involved in translation of collagen mRNAs, for example, STRAP, RHA, nonmuscle myosin and FKBP3^10^,^11^,^21^,^27^ and this domain is predicted to be unstructured. Therefore, phosphorylations in this domain may profoundly influence the interactions of LARP6 with these proteins and their recruitment into collagen biosynthetic pathway.

Our results are consistent with hypothesis that phosphorylation of S451 in LARP6 is an early phosphorylation event that is required for priming of other phosphorylation sites. The reasoning is based on several experiments. First, when S451 was mutated into alanine, the mutant LARP6 showed absence of multiple phospho-isoforms and not only one phosphorylation, as determined by disappearance of at least 3 phospho signals from 2DGE (Fig. 2b, panels 1 and 4). Second, when S451 was changed into phospho-mimetic mutation, S451D, the multiple phosphorylated isoforms were restored (Fig. 2b, panel 6). Third, when the C-terminal domain of LARP6 was expressed alone and the S451A mutation introduced, 3-4 phospho signals were abolished and the pI of the domain was increased by 2.2 pH units (Fig. 2c, panels 1 and 2). Fourth, when S451D mimic was introduced into the C-terminal domain, multiple phospho signals were restored (Fig. 2c, panel 3). The dependence of multiple phospho signals on phosphorylation of S451 suggests that this is the primary event in posttranslational modifications of LARP6.

Our efforts to identify the kinase involved in LARP6 phosphorylation pointed to the PI3K/Akt pathway (Fig. 2a). Identification of the Akt dependent phosphorylation of LARP6 at S451 was supported by the evidence obtained using *in vivo* and *in vitro* approaches, but the evidence that Akt directly phosphorylates S451 is still lacking. Bacterially expressed LARP6 could not be phosphorylated by purified Akt; although this could be due to a misfolded protein or to absence of some accessory factors, as described for other Akt substrates^40^,^41^,^42^. Therefore, we cannot conclude with certainty that S451 is the direct target of Akt. Regardless of this caveat, the importance of Akt mediated S451 phosphorylation in type I collagen synthesis is compelling. We found that CA Akt increases the relative amount of LARP6 highly phosphorylated, while the Akt inhibitor reduces the phosphorylation of LARP6 (Fig. 2b). However, Akt inhibitor has no effect on the phosphorylation status of S451A, suggesting that S451 is the major target of Akt (Fig. 2b).

We demonstrated that Akt can phosphorylate LARP6 *in vitro* when the protein is immunoprecipitated from cells. Immunoprecipitated LARP6 was phosphorylated in the absence of co-expressed active Akt or pure Akt added to the precipitate (Fig. 3a–d), suggesting that an endogenous Akt kinase was pulled down with LARP6. Co-immunoprecipitation experiments verified that LARP6 and Akt interact (Fig. 3e). The *in vitro* phosphorylation was prevented by Akt inhibitor, GSK-2141795 (Fig. 3c), suggesting that the kinase active in the immunoprecipitate is Akt. When the immunoprecipitation reactions were supplemented with active purified Akt, an increase in phosphorylation was observed, but only when wild type LARP6 was pulled down and not with pull down of S451A mutant (Fig. 3d), though this mutant interacts with Akt (Fig. 3e). Taken together, these results support the conclusion that Akt participates in phosphorylation of LARP6 and targets S451.

Amino acid sequence surrounding S451 does not conform to the Akt consensus sequence, RXRXXS/T^43^, and we do not know the mechanism by which Akt recognizes this region. However, other proteins that do not harbor the Akt consensus motif have been shown to be phosphorylated by Akt, such as RARα, IRAK1, and YB-1^44^,^45^,^46^.

In our study Akt inhibitor reduced predominantly type I collagen secretion into the cellular medium (Fig. 4a). Similar effect could be reproduced by overexpression of the S451A mutant (Fig. 4c). This clearly demonstrated the correlation between the phosphorylation of LARP6 at S451 and the ability of cells to excrete type I collagen. Overexpression of the S451D rescued collagen secretion after inhibition of Akt, suggesting that this mutant can override the inhibition of Akt signaling (Fig. 4h).

In biosynthesis of type I collagen the rate of translation of α1(I) and α2(I) polypeptides is synchronized to the rate of their posttranslational modifications and folding into a triple helix^47^. In some patients with osteogenesis imperfecta collagen polypeptides are hyper-modified due to mutations that impair the rate of folding. The hyper-modifications include excessive lysine hydroxylations and shift of the pI of collagen polypeptides into more acidic region^36^. Our previous work has demonstrated that translation of collagen polypeptides is impaired when LARP6 is knocked down, resulting in poor secretion and in acidic shift of the pI of α2(I) polypeptide^5^. When we analyzed the isoelectric point of α2 (I) polypeptide after Akt inhibiton, a fraction of molecules showed a shift to the more acidic pI (Fig. 4b). Moreover, similar acidic pI shift of the α2 (I) polypeptide could be reproduced by overexpression of S451A mutant (Fig. 4f). As alteration of pI of collagen polypeptides is a readout of impaired recognition and folding of type I collagen we concluded that lack of phosphorylation of LARP6 at S451 interferes with coordination of translation and folding of collagen polypeptides.

We also found that S451D mutant has shorter half life than wt LARP6 or S451A mutant (Fig. 5). As this mutant mimics phosphorylated LARP6, this implicates that activation of LARP6 by S451 phosphorylation may be accompanied by faster turnover of the protein. We could artificially prolong the half life of endogenous LARP6 by inhibiting proteasome for 6 hours (Fig. 5c). After proteasomal inhibition, the higher level of LARP6 was associated with slower secretion of type I collagen, although the intracellular levels of collagen polypeptides remained unchanged and secretion of fibronectin was unaffected. Although proteasome inhibition affects many cellular processes and interpretation of this result may not be simple, it is the first indication of the importance of regulating half life of LARP6.

Fibrosis is characterized by excessive synthesis of type I collagen. Several studies pointed to the role of PI3K/Akt in development of fibrosis^48^,^49^,^50^,^51^,^52^. We report for the first time that a potent Akt inhibitor, GSK-2141795, decreases type I collagen production. This inhibitor is currently in stage II clinical trials for treating a variety of solid cancers, including colon carcinoma, melanoma, ovarian carcinoma, breast cancer, and myeloma. We showed that GSK-2141795 inhibited secretion of type I collagen with EC_50_ of 150 nM (Fig. 6), which is similar to its IC_50_ for all three Akt isoforms. We postulate that potential anti-fibrotic activity of GSK-2141795 (and possibly of other Akt inhibitors) is due to the inhibition of LARP6 phosphorylation at the activating phosphorylation site, S451. Our finding has two practical implications: 1. at low doses, GSK-2141795 may be tried as treatment of fibrosis, 2. GSK-2141795 treatment of solid tumors may not only reduce the tumor growth, but also reduce the fibrous stroma surrounding the tumor, allowing easier penetration of other chemotherapeutics to the tumor cells.

In conclusion, we showed that phosphorylation of LARP6 plays an important role in regulating type I collagen biosynthesis. Primary phosphorylation of LARP6 at S451 requires Akt and Akt can be coimmunoprecipitated with LARP6. The phosphorylation of S451 is a prerequisite for phosphorylations of additional serines and for participation of LARP6 in regulating translation and subsequent folding and secretion of type I collagen. This findings support a novel mechanism for the anti-fibrotic effect of Akt inhibition through modulation of LARP6 phosphorylation.

## Methods

### Plasmid constructs and adenovirus construction

pcDNA3 vectors expressing human LARP6 tagged with HA tag at the amino terminus (HA-LARP6) and the HA-tagged carboxyl terminal domain (HA-CTER) of LARP6 have been described previously^17^. Site directed mutagenesis of the single amino acids was done by QuickChange mutagenesis kit (Stratagene, 200523-5), according to the instructions from the manufacturer. The identity of all mutations was verified by sequencing and their expression by Western blotting. Plasmid expressing constitutive active Akt (myr Akt delta4-129, CA Akt) was from Addgene (10841)^35^.

Adenoviruses were constructed by re-cloning the full-length wild type HA-LARP6 and HA-tagged LARP6 carrying single point mutations from pcDNA3 vectors into pAd-CMV-Track vector, followed by recombination with pAdEasy vector in BJ5183 E. coli cells^53^. Adenovirus were propagated in HEK293 cells and purified by cesium chloride density gradient centrifugation. The viruses expressed both LARP6 and GFP, which was encoded by an independent transcription unit^53^. Expression of GFP was used as a marker to monitor the efficiency of viral transduction.

### Chemicals and antibodies

PDK1 inhibitor was from Calbiochem (124015). LY294002 was purchased from Cayman Chemical (154447-36-6). U0126 was from Calbiochem (662005). GSK-2141795 was from Activebiochem (A-1504). Pure active Akt enzyme was purchased from SignalChem (A16-10G). Calf intestinal alkaline phosphatase (CIP) was from New England Biolabs (M0290S). MG132 was from VWR International (89161566). Antibodies used were: anti LARP6 antibody from Abnova (H00055323-B01P), anti-HA antibody from Sigma-Aldrich (H9658), anti-collagen α1 (I) antibody from Rockland (600-401-103), anti-collagen α2 (I) antibody from Santa Cruz Biotechnology (sc-8786), anti-fibronectin antibody from BD Transduction Laboratories (610077), anti-β-actin antibody from abcam (ab8227), and anti-pan Akt antibody from Cell Signaling (4691).

### Cells and transfections

HEK293 cells and human lung fibroblasts (HLFs) immortalized by expression of telomerase reverse transcriptase^54^ were grown under standard conditions. Transfections were done in 6-well plates with 1 μg of plasmid using TransIT-293 transfection reagent (Mirus, MIR2700). The cells were harvested 48 hours after the transfections. The inhibitors were added at the indicated concentrations for 12 hours prior to cell harvesting for analysis of LARP6 phosphorylation by two-dimensional gel electrophoresis (2DGE).

Transduction of human lung fibroblasts with adenoviruses was done at multiplicity of infection (MOI) of 500. With this MOI, 95-100% of the cells were transduced, as visualized by the expression of GFP. 48 hours after the transduction, cell extracts were made and analyzed by Western blotting or 2DGE. The inhibitors were added at the indicated concentrations for 12 hours prior to cell harvesting for analysis of collagen expression and for 6 hours when LARP6 phosphorylation was analyzed by 1DGE. For estimation of half life of LARP6, HLFs were transduced with adenovirus for 6 hours and treated with cycloheximide (100 μg/ml) for 0, 6, 12, and 18 hours and analyzed by Western blotting.

### Real-time PCR analysis

RNA was extracted from cultured HLFs using Trizol reagent (Invitrogen) according to the manufacturer’s instructions. 1 μg of RNA was used to prepare cDNA using the Superscript First Strand Synthesis System for RT-PCR (Invitrogen), according to the manufacturer’s instructions. 5 μl of 10-fold-diluted cDNA was used in SYBR Green qPCR assay (Applied Biosystems) on LightCycler 480 Real-Time RCR instrument (Roche Applied Sciences). The primers used for PCR amplification were shown in Table 2. Expression of test genes was normalized to that of β-actin. The statistical significance of the qPCR results was calculated using Student’s t test, with P values of <0.05 as significant. The results are presented as mean ± standard deviation (SD).

### Western blotting analysis

For one dimensional Western blotting analysis of LARP6, HLFs were treated with DMSO or kinase inhibitors, PDK1 inhibitor (4 μM), LY294002 (100 μM), U0126 (10 μM), and GSK-2141795 (0.1 μM) for 2 hours, adenovirus overexpressing wt HA-LARP6 or S451A LARP6 mutant was added and incubated for additional 4 hours. When needed, HLFs were treated with MG132 (8 μM) for 3 and 6 hours. The cells were lysed in Tris-HCl (50 mM), pH 7.5, NaCl (150 mM), 1% NP-40, 0.5% Sodium Deoxycholate, 0.1% SDS, Dithiothreitol (1 mM) and protein concentrations were estimated with the Bradford assay (Biorad, 500-0006), with bovine serum albumin (BSA) as the standard. 40 μg of total cellular protein was typically used for Western blotting analysis.

For Western blotting analysis of cellular medium proteins, equal numbers of cells were seeded in 6-well plates and after 24–48 hours the cells were washed three times with serum free medium (washing with serum free medium was essential, as serum contains substantial amounts of collagen and fibronectin). 600 μl of serum free medium was then added to the cells and incubation continued for 3 hours. The medium was collected and an aliquot directly analyzed by Western blotting.

Western blot signals were quantified using ImageJ software using the results of at least three independent experiments (n = 3). The level of LARP6 was normalized to the level of β-actin in each sample. The level of collagen α1 (I) (COL1A1) and collagen α2 (I) (COL1A2) polypeptides was normalized to the level of β-actin (intracellular) or fibronectin (cellular medium). The statistical significance of the LARP6 and collagen results was calculated using Student’s t test, with P values of <0.05 as significant. The results are presented as mean ± SD.

### Two-dimensional gel electrophoresis

Cells were lysed in 0.5% NP-40, Tris-HCl (50 mM), pH 7.5, NaCl (150 mM), phenylmethylsulfonyl fluoride (170 μg/ml), 1 × proteinase inhibitor, sodium fluoride (50 mM), β-glycerophosphate (5 mM) and sodium orthovanadate (1 mM) were added when phosphorylation of LARP6 was analyzed. Proteins were precipitated with 9 volumes of 100% ethanol and recovered by centrifugation at 2,284 × g for 10 minutes at 4 °C^55^. The protein pellet was solubilized in rehydration buffer (Urea (7 M), Thiourea (2 M), 2% CHAPS, 0.8% Ampholytes, Dithiothreitol (65 mM), bromophenol blue) for 1 hour at room temperature, and loaded onto Immobiline Dry Strip strips (7 cm, pH 3 to 10, GE Healthcare, 17-6001-11). The first-dimension separation was on Ettan IPGphor 3 instrument (GE Healthcare), according to standard protocol^56^,^57^. The second-dimension separation was done by laying strips onto 7.5% SDS PAGE, followed by Western blotting. Immobilized strips showed slight batch to batch variations in the ampholyte distribution, so only the samples run on the same batch of strips were directly compared.

### Immunoprecipitations

Cells were lysed in 500 μl of Tris-HCl (50 mM), pH 7.5, NaCl (150 mM), 0.5% NP-40, Dithiothreitol (1 mM), phenylmethylsulfonyl fluoride (170 μg/ml), 1 × proteinase inhibitors and cleared lysate was incubated with 1 μg of antibody for 3 hours at 4 °C. 30 μl of equilibrated protein A/G-agarose beads (Santa Cruz Biotechnology) was added, and incubation continued for 1 hour. The beads were washed three times with lysis buffer and loaded onto SDS PAGE gels followed by Western blotting.

### Mass Spectrometry

HA-LARP6 was expressed and immunoprecipitated from HLFs in presence of phosphatase inhibitors. The immunoprecipitated protein was resolved on SDS PAGE gel and stained with GelCode Blue Stain Reagent (Thermo Scientific, 24590). The HA-LARP6 band was excised and in-gel trypsin digest was done using ProteoExtract All-in-One Trypsin Digestion Kit (Calbiochem, 650212) for 2 hours at 37 °C with shaking. Peptides were eluted in 50 μl 0.1% formic acid, separated on LCMS and the LC eluent was directly nano-sprayed into an LTQ Orbitrap Velos mass spectrometer (Thermo Scientific). The MS data were acquired using the following parameters: 10 data-dependent collisional-induced-dissociation (CID) MS/MS scans per full scan (400 to 2000 m/z) at a mass resolution for MS1 of 60000, minimum signal required to trigger MS2 was 500, MS mass range 0 to 1000000 and dynamic exclusion enabled with following parameters: Repeat count:1, Repeat Duration: 30.00, exclusion list size: 500, exclusion duration: 60.00, exclusion mass width relative to low and high mass: 10 ppm. All measurements were performed at room temperature and three technical replicates per sample were run to allow for statistical comparisons. The raw files were analyzed using Proteome Discoverer (version 1.4) software package with SequestHT and Mascot search nodes using *Homo sapiens* specific FASTA database and the Percolator peptide validator. Phosphorylation was detected by both SequestHT and Mascot and was verified by inbuilt phosphoRS node in proteome discoverer. Scaffold (version Scaffold_4.3.4, Proteome Software Inc., Portland, OR) was used to validate MS/MS-based peptide and protein identifications. Peptide identifications were accepted if they could be established at greater than 95.0% probability by the Scaffold Local FDR algorithm.

### *In vitro* kinase Assay

HLFs were transduced with adenovirus encoding wt HA-LARP6 and HA-LARP6 S451A mutant. After 48 hours the cell lysate was prepared and subjected to immunoprecipitation with anti-HA antibody. The immunoprecipitate was washed three times and resuspended in 30 μl of kinase reaction buffer (Τris-HCl (50 mΜ), pH 7.5, MgCl_2_ (10 mM), sodium fluoride (5 mM), β-glycerophosphate (5 mM), sodium orthovanadate (5 mM), ATP (50 μM), [γ-^32^P]ATP (0.5 μCi). GSK-2141795 at 0.1 μM (diluted in kinase reaction buffer) was preincubated with the immunoprecipitate for 30 minutes at 37 °C prior to addition of the kinase buffer. When the reaction was supplemented with exogenous Akt, 200 ng of pure, active Akt protein (SignalChem, A16-10G) was added to the kinase buffer. The kinase reactions were incubated at 37 °C for 1 hour, stopped by adding 6 × SDS protein loading buffer, heated and run on SDS polyacrylamide gel. The gel was dried and subjected to autoradiography.

## Additional Information

**How to cite this article**: Zhang, Y. and Stefanovic, B. Akt mediated phosphorylation of LARP6; critical step in biosynthesis of type I collagen. *Sci. Rep.* **6**, 22597; doi: 10.1038/srep22597 (2016).

## Figures and Tables

**Table 1:** Identification of LARP6 phosphorylation sites by Mass Spectrometry.

Position	Target	Modification	PTM Score	Peptide Confidence	Sequence Motif
56	S	Phospho	100	High	LSPGWG**S**ASEEEP
58	S	Phospho	100	High	PGWGSA**S**EEEPSR
396	S	Phospho	100	High	KGVSRK**S**PLAEEG
421	S	Phospho	99.8	High	KCMDYS**S**DSSVTP
447	S	Phospho	98.6	High	MGTQEK**S**PGTSPL
451	S	Phospho	97.3	High	EKSPGT**S**PLLSRK
409	S	Phospho	96.4	High	RLNCST**S**PEIFRK
348	S	Phospho	92.7	High	PESNPT**S**PMAGR

**Table 2:** Real-time PCR primers used.

Gene names	Primer sequences
human collagen α1(I)	F: 5′-AGAGGCGAAGGCAACAGTCG-3′
	R: 5′-GCAGGGCCAATGTCTAGTCC-3′
human collagen α2(I)	F: 5′-CTTCGTGCCTAGCAACATGC-3′
	R: 5′-TCAACACCATCTCTGCCTCG-3′
human β-actin	F: 5′-GTGCGTGACATTAAGGAGAAG-3′
	R: 5′-GAAGGTAGTTTCGTGGATGCC-3′
